# Evidence of a Cooler Continental Climate in East China during the Warm Early Cenozoic

**DOI:** 10.1371/journal.pone.0155507

**Published:** 2016-05-19

**Authors:** Qian-Qian Zhang, Thierry Smith, Jian Yang, Cheng-Sen Li

**Affiliations:** 1 State Key Laboratory of Systematic and Evolutionary Botany, Institute of Botany, Chinese Academy of Sciences, Xiangshan, Beijing, P. R. China; 2 Institute of Geomechanics, Chinese Academy of Geological Sciences, Beijing, P. R. China; 3 Direction Earth and History of Life, Royal Belgian Institute of Natural Sciences, Brussels, Belgium; Agharkar Research Institute, INDIA

## Abstract

The early Cenozoic was characterized by a very warm climate especially during the Early Eocene. To understand climatic changes in eastern Asia, we reconstructed the Early Eocene vegetation and climate based on palynological data of a borehole from Wutu coal mine, East China and evaluated the climatic differences between eastern Asia and Central Europe. The Wutu palynological assemblages indicated a warm temperate vegetation succession comprising mixed needle- and broad-leaved forests. Three periods of vegetation succession over time were recognized. The changes of palynomorph relative abundance indicated that period 1 was warm and humid, period 2 was relatively warmer and wetter, and period 3 was cooler and drier again. The climatic parameters estimated by the coexistence approach (CA) suggested that the Early Eocene climate in Wutu was warmer and wetter. Mean annual temperature (MAT) was approximately 16°C and mean annual precipitation (MAP) was 800–1400 mm. Comparison of the Early Eocene climatic parameters of Wutu with those of 39 other fossil floras of different age in East China, reveals that 1) the climate became gradually cooler during the last 65 million years, with MAT dropping by 9.3°C. This cooling trend coincided with the ocean temperature changes but with weaker amplitude; 2) the Early Eocene climate was cooler in East China than in Central Europe; 3) the cooling trend in East China (MAT dropped by 6.9°C) was gentler than in Central Europe (MAT dropped by 13°C) during the last 45 million years.

## Introduction

The early Cenozoic climate was characterized by a much warmer mean global temperature than today, the extreme case being the Early Eocene Climatic Optimum (EECO), 53–51 million years ago, when concentrations of greenhouse gases were high and global temperature reached a long-term maximum [1]. The Early Eocene is also marked by the rapid evolution and diversity of early modern plants and vertebrates with important intra- and intercontinental dispersals starting during the Paleocene-Eocene Thermal Maximum, about 55 million years ago [2, 3]. The response of vegetation succession to the Early Eocene climatic changes in the Northern Hemisphere has been extensively studied in North America and Europe [4–11], but it is still poorly understood in Asia. Only a series of quantitative researches on Paleogene climate in East China were carried out in recent years [12–16]. In this study, China would thus provide important information for the third Northern continent.

The Early Eocene Wutu Formation in Shandong Province, Eastern China is one of the most important deposits in Asia for the understanding of early modern mammal evolution. Since the 1930s, this deposit has indeed yielded an intriguingly mixed fauna of archaic and early modern mammals [17, 18]. More recently, early modern plants such as the oldest Asian Nymphaeaceae and earliest record of *Prunus* have been found in the Wutu deposit [19, 20] that corresponds to one of the warmest time periods of the Cenozoic. Therefore, Wutu occupied a key position in the palaeogeography of Asia especially for biotic exchanges with the rest of Asia and with North America.

Previous investigations at Wutu have indicated a potentially wide palynostratigraphical distribution [21, 22]. Therefore, we analyzed the palynological samples of a 300 m borehole drilled in the Wutu Formation in order to reconstruct the Early Eocene vegetation succession and climatic changes in Wutu by the coexistence approach method. Furthermore, in combination with the prior quantitative researches, we explored the Cenozoic climatic changes in East China.

## Materials and Methods

**Fig 1:**
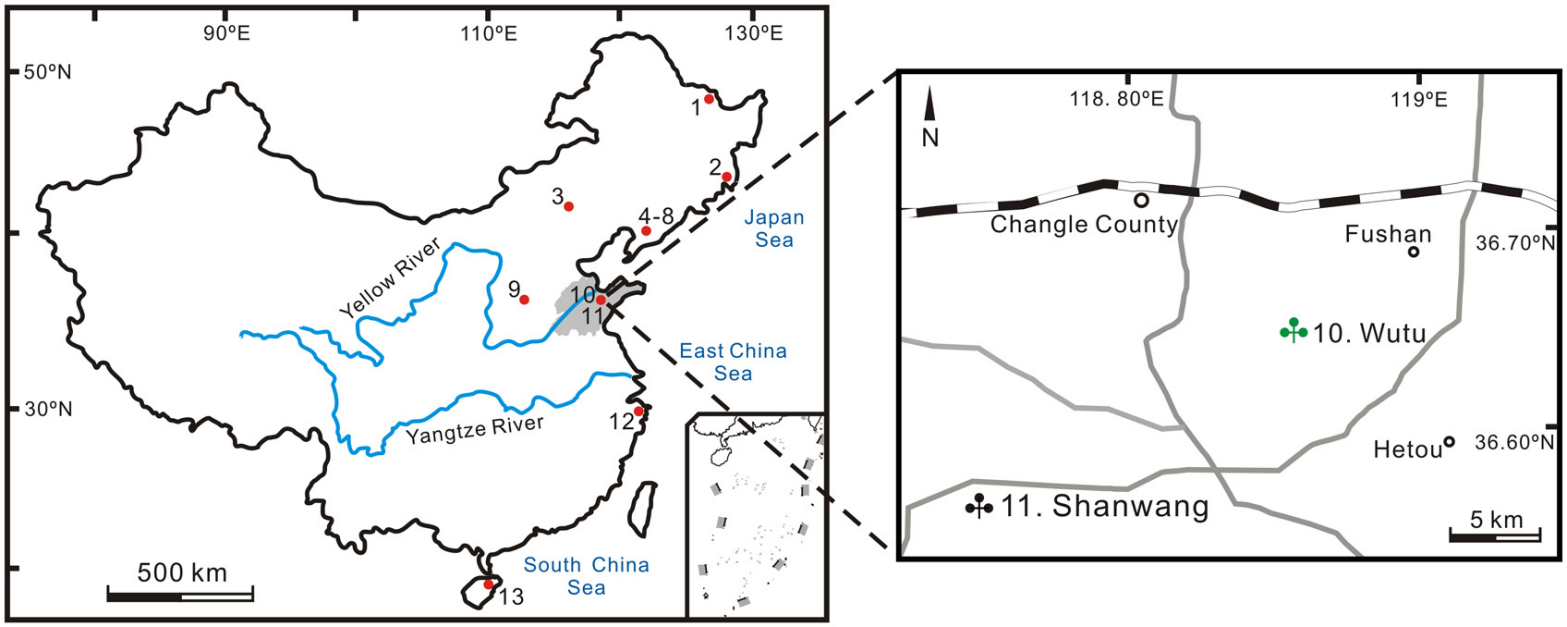
Maps showing the position of the Wutu coal mine and other localities of East China where prior quantitative researches have been investigated. (modified from Shandong Provincial Institute of Land Surveying and Mapping [48].) 1. Wuyun, 2. Huanan, 3. Yilan, 4. Jidong, 5. Hualin, 6. Mudanjiang, 7. Shulan, 8. Dunhua, 9. Erlian, 10. Huadian, 11. Hunchun, 12. Weichang, 13. Fushun, 14. Shangdou, 15. Huanghua, 16. Zhangcun, 17. Jiyang, 18. Bozhong, 19. Wutu, 20. Shanwang, 21. Lantian, 22. Tianchang, 23. Zhoukou, 24. Du’ao, 25. Changchang.

**Fig 2:**
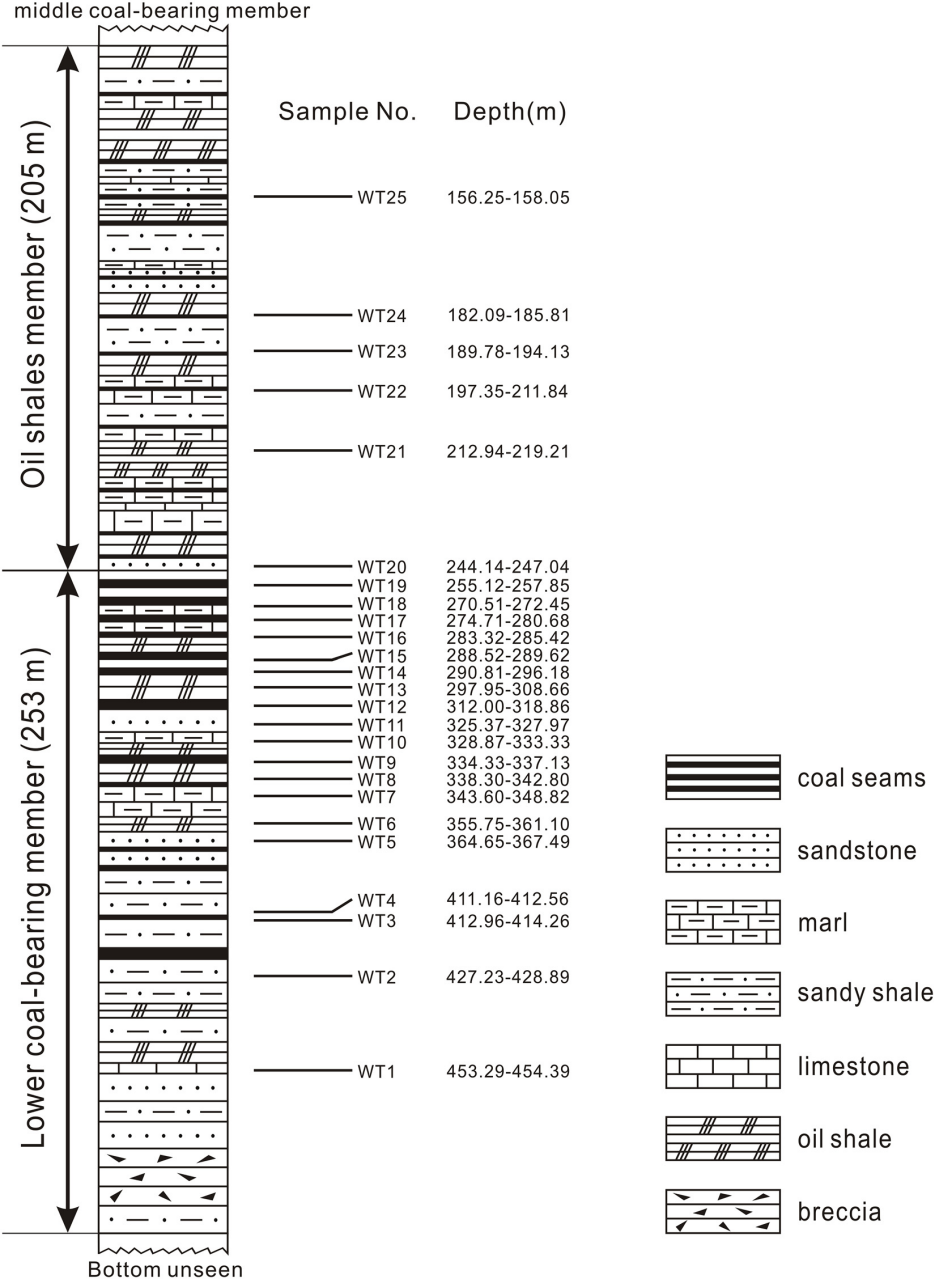
Measured stratigraphic sequence of the Wutu borehole.

The Wutu coal mine (36°39′N, 118°55′E) is located near the town of Wutu, Linqu County, Shandong Province, East China (Fig 1). The sediments in the coal mine belong to the Wutu Formation which is late Early Eocene in age based on the seed-eating Carpolestidae mammals [23] and confirmed by a diversified mammal association (e.g. *Preonictis youngi*, *Zodiocyon zetesios*, *Pappomoropus taishanensis*, *Chowliia laoshanensis*, *Homogalax wutuensis*, *Wutuhyus primiveris*) [18]. The Wutu Formation consists of four members [24], from bottom to top: the lower coal-bearing member, the oil shale member, the middle coal-bearing member, and the upper coal-bearing member. Twenty-five palynological samples were collected from the lower coal-bearing and oil shale members (Fig 2). The borehole was taken several years ago from Wutu coal mine during a geology survey. After a series of geological researches, there were only 25 left samples for the palynological study. In this study, the specimen collection did not involve endangered or protected species, so specific permission was not required.

**Fig 3:**
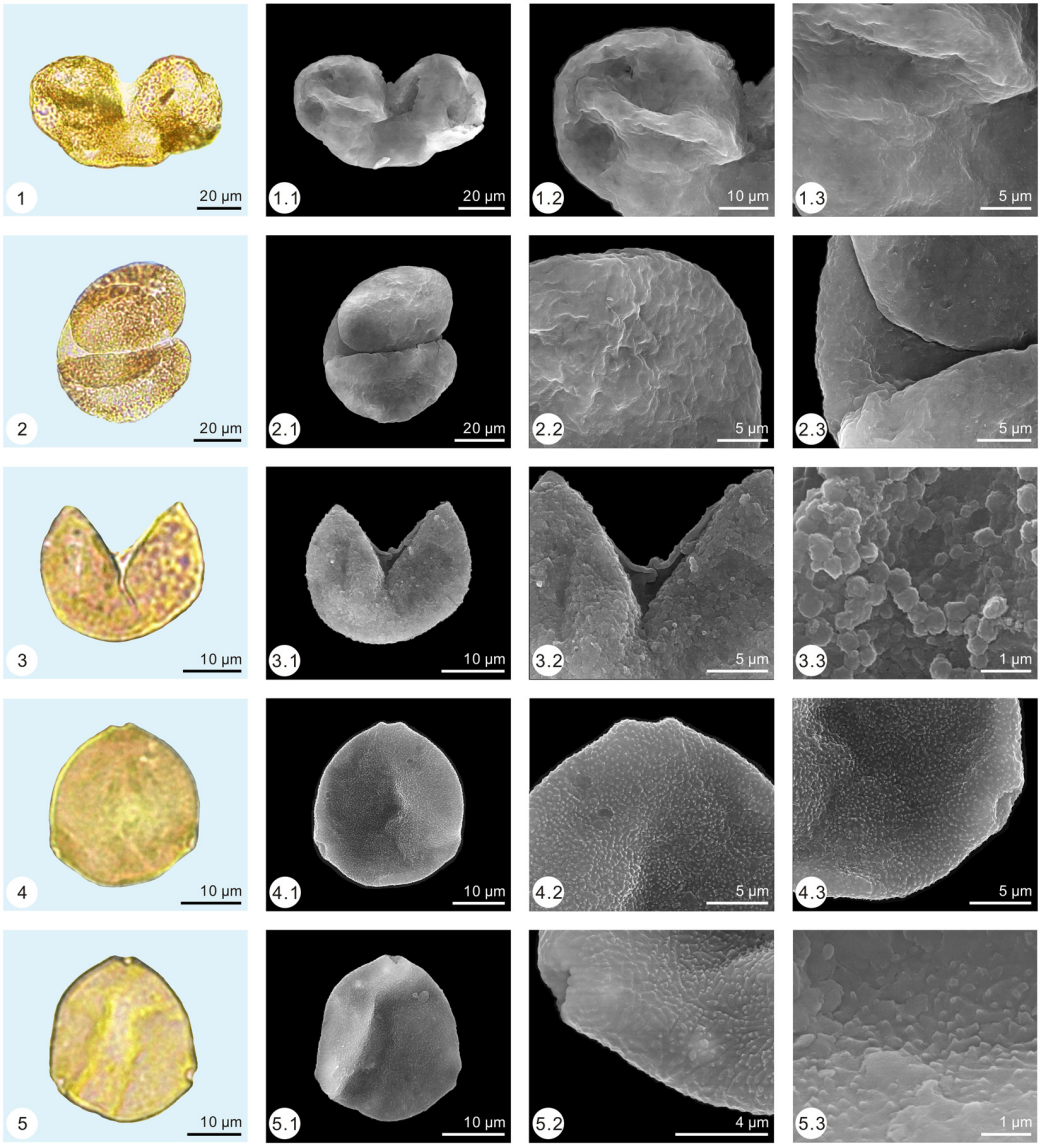
Pollen assemblage from the Early Eocene of Wutu. 1. *Pinuspollenites* (1.2. show details of air sac; 1.3. show details of the body), 2. *Piceapollis*, 3. *Taxodiaceaepollenites*, 4–5. *Momipites coryloides*.

**Fig 4:**
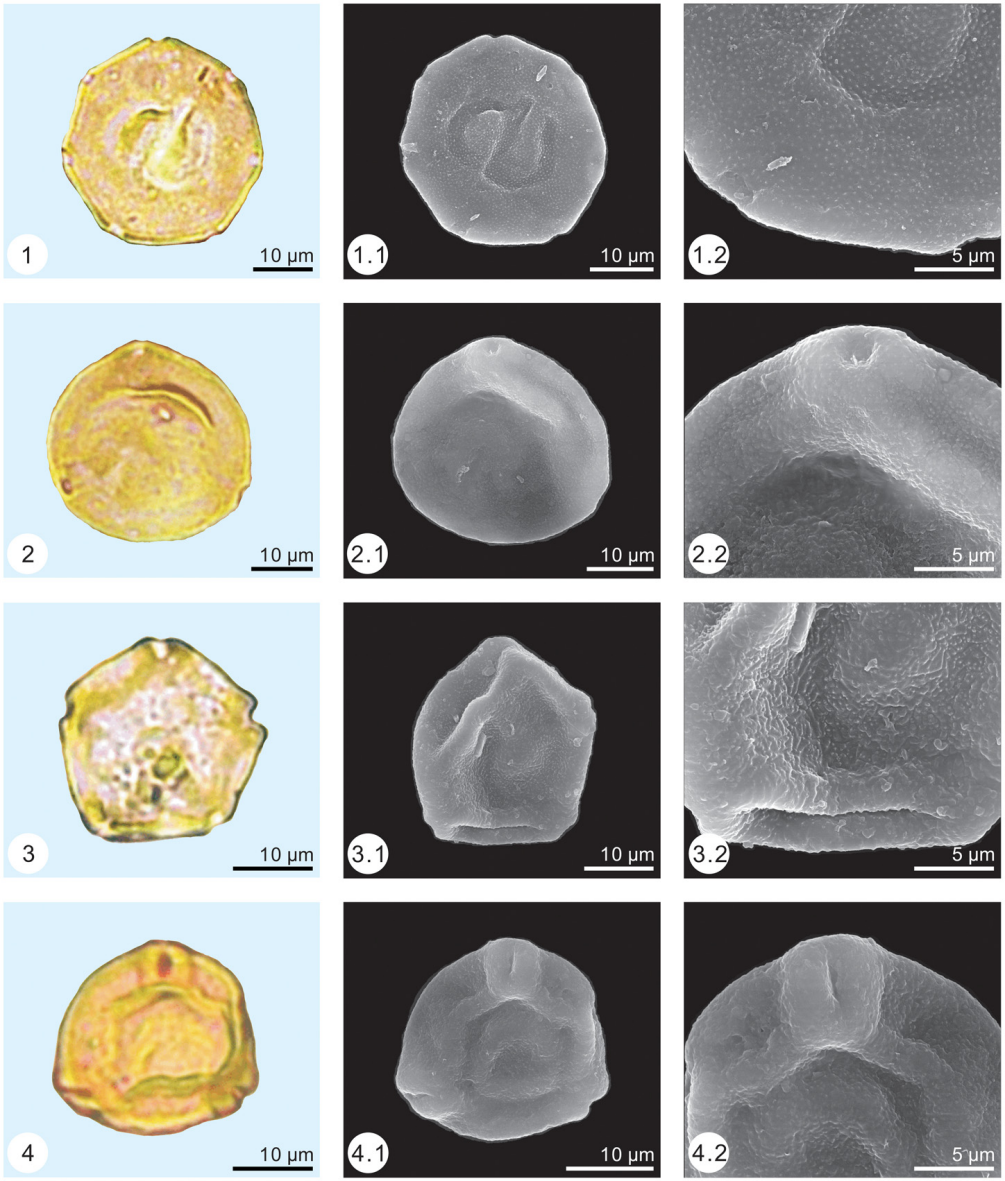
Pollen assemblage from the Early Eocene of Wutu. 1. *Juglanspollenites*, 2. *Caryapollenites*, 3. *Alnipollenites*, 4. *Betulaceoipollenites*.

**Fig 5:**
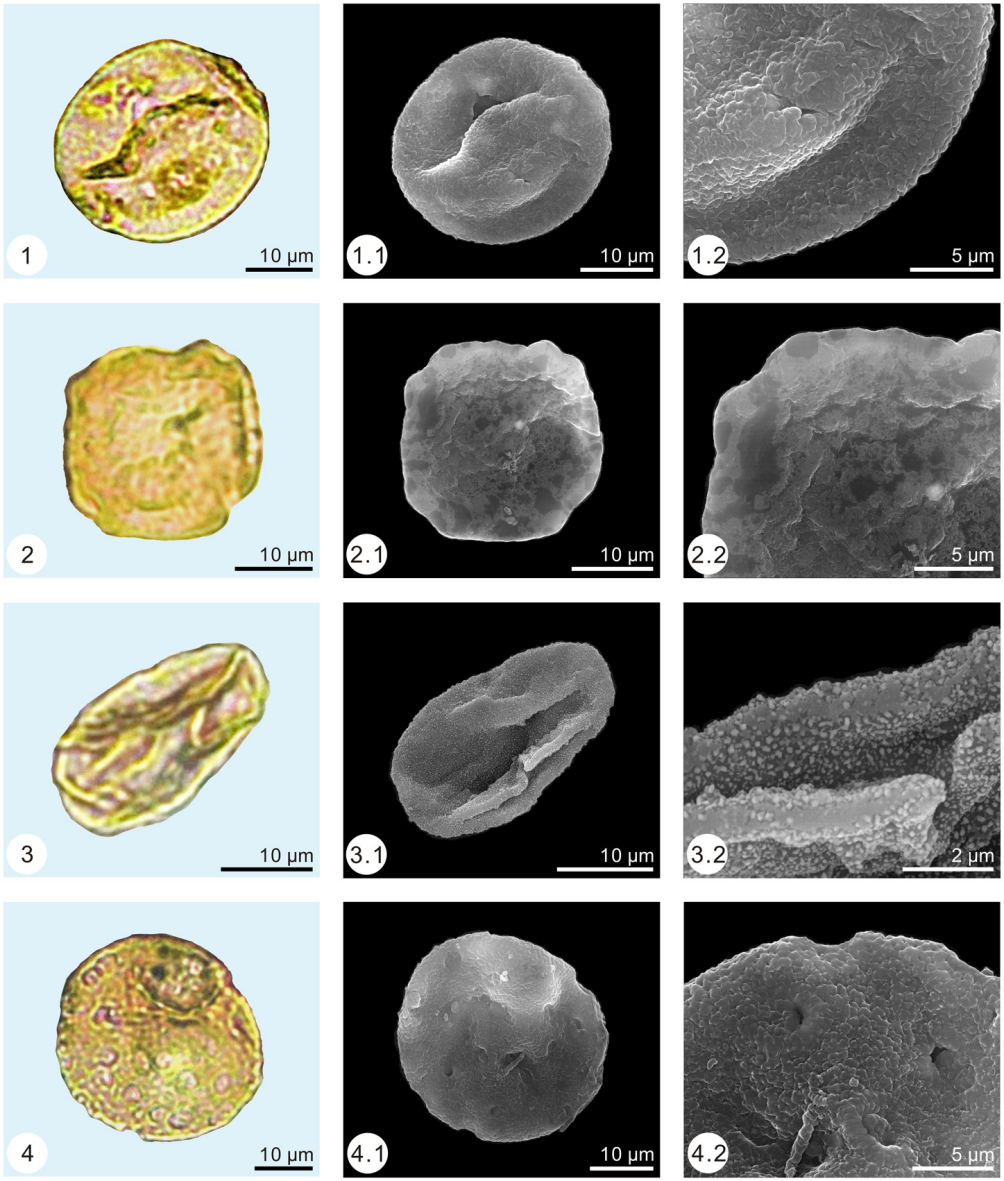
Pollen assemblage from the Early Eocene of Wutu. 1. *Graminidites*, 2. *Ulmipollenites*, 3. *Castanopsis*, 4. *Chenopodipollis*.

The palynological samples were treated by the method of Heavy Liquid Separation (density = 2.1 g/ml) [25, 26]. Palynomorph grains were found in all samples. More than 100 grains (113–766) were found in 11 samples (samples number: 1, 2, 7, 10, 11, 18, 19, 21, 22, 24 and 25), while less than 100 grains (1–59) were found in the other 14 samples. The low amounts of palynomorph grains may be caused by taphonomy condition. More than 2,800 palynomorph grains were observed under a Leica DM 2500 light microscope and identified based on the classification of Song [27]. By applying the single-grain technique [28], palynomorph grains were photographed with a Zeiss Axioplan 2 light microscope and a FEI Quanta 200 environmental scanning electron microscope (Figs 3–5).

**Fig 6:**
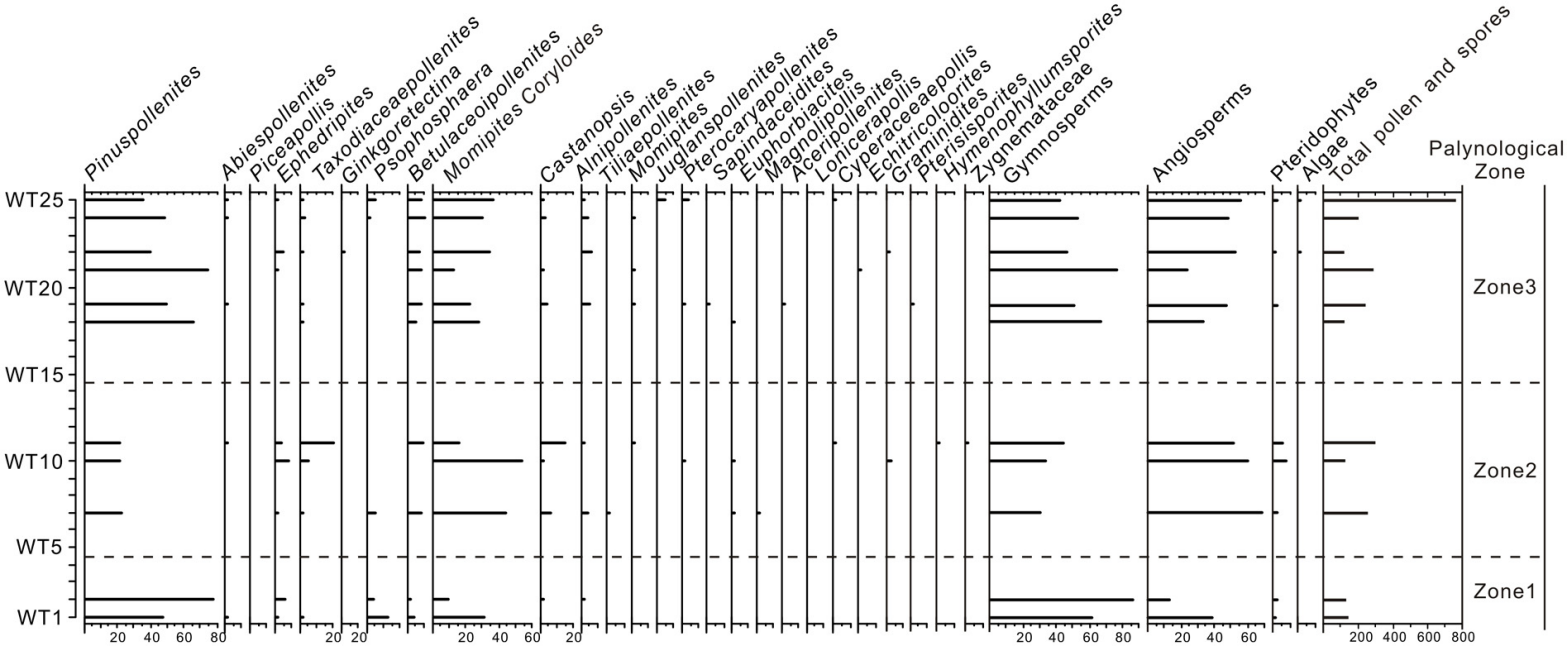
Pollen diagram showing percentage values of the main taxa from Wutu.

The palynomorph relative abundance (RA) of a taxon was calculated by the following equation: RA = N/Nt, where N is the pollen/spore number of a taxon and Nt is the total pollen/spore number of all taxa combined in the pool of samples [29, 30]. TILIA and TILIAGRAPH softwares were used to construct the pollen diagram (Fig 6).

In this work, according to the temperature requirements of their nearest living relatives (NLRs), the palynomorph taxa were divided into seven groups: megathermic, mega-mesothermic, mesothermic, meso-microthermic, microthermic, nonsignificant elements and herbs and/or shrubs [29–32]. We used the CA [33–35] in the quantitative reconstruction of the palaeoclimate in Wutu. The basic principle of CA is the assumption that the climatic requirement of a fossil taxon was similar to that of its NLR. The climatic parameters in the modern geographic distributions of all NLRs were compared [36], and then the coexistence intervals of the climatic tolerances of the fossil taxa were obtained. The modern climatic parameters of NLRs used in CA were extracted from Surface Meteorological Data of China [37]. Meanwhile, the MAT values of NLRs from the Palaeoflora Database were also adopted to obtain the climatic parameters [38]. It resulted in seven palaeoclimatic parameters for Wutu: MAT = the mean annual temperature, MWMT = the mean temperature of the warmest month, MCMT = the mean temperature of the coldest month, DT = the difference in temperature between the coldest and warmest months, MAP = the mean annual precipitation, MMaP = the mean maximum monthly precipitation and MMiP = the mean minimum monthly precipitation.

Based on the study of modern floras, Grimm and Denk [39] referred to the CA as a less reliable approach for climate reconstructions. However, the CA was earlier tested with modern vegetation [35, 40], and was applied on Cenozoic floras of Central Europe to obtain the continuous continental climate curve which closely correlated with the evolution of marine temperature [41]. According to our works by using the CA in the last more than 10 years, we found that this method needs to be improved to some extent, but it is still very useful for reconstructing the Cenozoic climatic changes in East China.

## Results

### Palynological assemblages

Forty-seven different palynomorphs were identified in the Wutu section, consisting of 28 angiosperms (48.36%), 7 gymnosperms (49.51%), 10 pteridophytes (1.99%) and 2 algae (0.14%) (Table A in S1 File). The angiosperms were assigned to 20 families and one to ambiguous type. The gymnosperms belonged to 4 families and one to ambiguous type. The pteridophytes belonged to 8 families and 2 to ambiguous type.

Of the 25 collected samples 11 were rich enough to perform the TILIA analysis recognizing three zones from bottom to top, based on the RA (Fig 6).

*Zone 1* (sample numbers WT1-WT4). 15 types of palynomorphs were found, including 6 angiosperms (30.82%), 5 gymnosperms (67.38%) and 4 pteridophytes (1.79%) (Table A in S1 File).

No megathermic element appeared in this zone (Table B in S1 File). Two mega-mesothermic elements (*Taxodiaceaepollenites* and *Castanopsis*), 4 mesothermic elements (*Alnipollenites*, *Betulaceoipollenites*, *Momipites* and *Caryapollenites*), one meso-microthermic element (*Pinuspollenites*), one microthermic element (*Abiespollenites*), and one kind of herbs and/or shrubs (*Ephedripites*) were detected.

The RA of megathermic and mega-mesothermic elements represents a total of 1.79%. The RA of microthermic and meso-microthermic elements is 56.27% (Table B in S1 File).

*Zone 2* (sample numbers WT5-WT14). In this zone, 35 palynomorphs comprised 21 angiosperms (60.95%), 5 gymnosperms (35.22%), 8 pteridophytes (3.69%) and one alga (0.13%). Compared with Zone 1, the RA of gymnosperm pollen decreased, while those of angiosperm pollen and pteridophytic spores increased (Table A in S1 File).

One megathermic element (*Palmaepollenites*), 5 mega-mesothermic elements (*Taxodiaceaepollenites*, *Castanopsis*, Bignoniaceae, *Euphorbiacites* and *Magnolipollis*), 10 mesothermic elements (*Momipites coryloides*, *Alnipollenites*, *Pterocaryapollenites*, *Caryapollenites*, *Juglanspollenites*, *Momipites*, *Ulmipollenites*, *Betulaceoipollenites*, *Lonicerapollis*, *Tiliaepollenites*), one meso-microthermic element (*Pinuspollenites*), one microthermic element (*Abiespollenites*), one nonsignificant element (*Tricolporopollenites rosaeformis*), and 5 kinds of herbs and/or shrubs (*Ephedripites*, *Graminidites*, *Artemisiaepollenites*, *Corsinipollenites*, *Cyperaceaepollis*) were present in this zone (Table B in S1 File).

In comparison with Zone 1, the average RA of megathermic and mega-mesothermic elements increased to 17.28%, while those of microthermic and meso-microthermic elements decreased to 21.64% (Table B in S1 File).

*Zone 3* (sample numbers WT15-WT25). 35 palynomorphs were recorded, including 21 angiosperms (45.85%), 7 gymnosperms (52.68%) and 6 pteridophytes (1.31%) and 2 algae (0.16%). Compared with Zone 2, the gymnosperms, replacing angiosperms, dominated in the assemblage again (Table A in S1 File).

Two megathermic elements (*Sapindaceidites* and *Palmaepollenites*), 3 mega-mesothermic elements (*Taxodiaceaepollenites*, *Castanopsis* and *Euphorbiacites*), 11 mesothermic elements (*Momipites coryloides*, *Alnipollenites*, *Betulaceoipollenites*, *Pterocaryapollenites*, *Ilexpollenites*, *Caryapollenites*, *Juglanspollenites*, *Momipites*, *Quercoidites*, *Aceripollenites*, *Ginkgoretectina*), one meso-microthermic element (*Pinuspollenites*), 2 microthermic elements (*Abiespollenites* and *Piceapollis*), and 7 kinds of herbs and/or shrubs (*Ephedripites*, *Graminidites*, *Cyperaceaepollis*, *Echitricoloorites*, *Potamogetonacidites*, *Umbelliferaepites*, *Chenopodipollis*) were detected in this zone (Table B in S1 File).

In comparison with Zone 2, the RA of megathermic and mega-mesothermic elements decreased to 3.06%, while those of microthermic and meso-microthermic elements increased to 48.06% (Table B in S1 File).

### Palaeovegetation succession

The whole palynological assemblage suggests that the Early Eocene vegetation of Wutu was composed of a mixed deciduous broad-leaved and coniferous forest growing under warm temperate condition. The co-occurence of megathermic and microthermic elements might suggest the presence of zonal vegetation in the hills nearby. The megatherm and mega-mesotherm grew sparsely in the valleys and/or basins. The mesotherm probably grew on the hillsides at low altitudes. The microtherm and meso-microtherm were likely to occupy the hills at higher altitudes.

**Fig 7:**
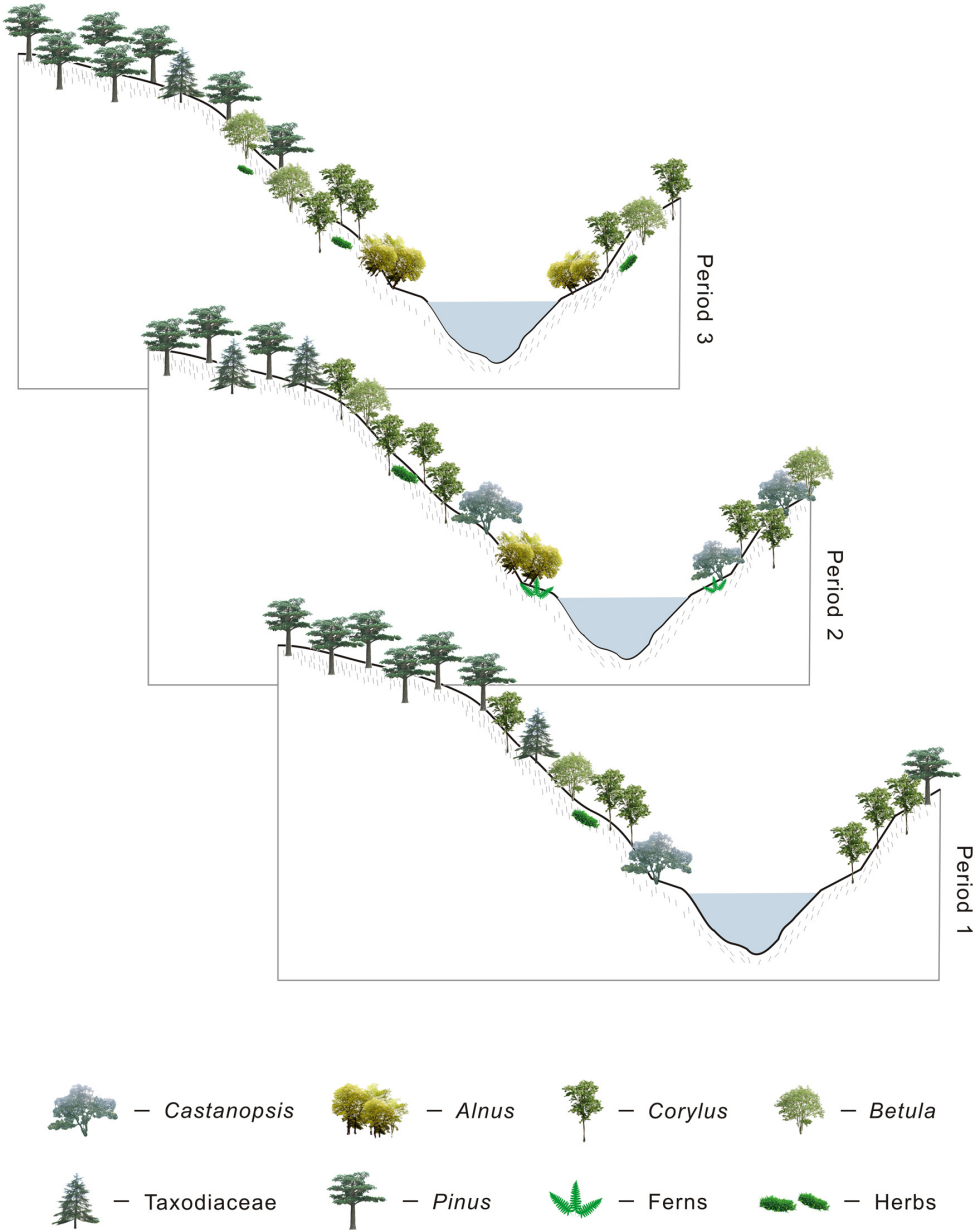
Reconstruction of the Early Eocene vegetation succession in Wutu based on the pollen diagram.

Based on the palynological data from the three zones, the vegetation succession in Wutu during the Early Eocene is divided into three periods and described as follows (Fig 7):

**Period 1** (Zone 1): The conifers dominated the mixed needle- and broad-leaved forest during this period and the vegetation was characterized by low plant diversity.

Mesothermic and meso-microthermic elements such as *Pinus*, *Betula* and *Corylus* dominated the forest. Thermophilous elements (Taxodiaceae and *Castanopsis*) and psychrophilous elements (*Pinus*, *Abies*) were present. Hygrophilous elements such as *Alnus* and Taxodiaceae grew sparsely around the paleolake or surrounding wetland. Xerophilous element (*Ephedra*) may be occurred on the dry slopes.

**Period 2** (Zone 2): In contrast to the low plant diversity during the Period 1, the diversity of plant increased. Instead of conifers, broad-leaved trees such as *Corylus* and *Betula* predominated in the mixed needle- and broad-leaved forest during this period.

In comparison with the Period 1, the diversity and RA of thermophilous elements (Taxodiaceae, *Castanopsis*, Euphorbiaceae, Magnoliaceae, Arecaceae and Bignoniaceae) increased, while the RA of psychrophilous elements (*Pinus*, *Abies*) decreased sharply. The hygrophilous elements (e.g. *Alnus*, Taxodiaceae) increased dramatically, with xerophilous elements (*Ephedra* and *Artemisia*) growing sparsely in the herbaceous layer of the forest.

**Period 3** (Zone 3): During this period, conifers increased dramatically and dominated the forest again. *Corylus* and *Betula* remained as the dominant broad-leaved type elements but their percentages decreased.

In comparison with the Period 2, thermophilous elements like Euphorbiaceae and Arecaceae decreased, while the psychrophilous elements such as *Picea* and *Abies* increased dramatically. Hygrophilous elements (e.g. *Alnus*, Taxodiaceae) decreased, indicating a dryer condition.

### Palaeoclimate and climatic fluctuations in Wutu

Based on the NLRs of 33 spermatophyte taxa from the whole pollen assemblage (Table C in S1 File), seven climate parameters of the Wutu section were estimated by applying the CA method (Figure A in S1 File). They were MAT = 11.5–20.8°C, delimited by Arecaceae and *Tilia*; MWMT = 19.8–28.0°C, delimited by *Carya* and Umbelliferae/Asteraceae; MCMT = -0.2–5.9°C, delimited by Arecaceae and *Ephedra*; DT = 12.3–26.0°C, delimited by *Picea* and Arecaceae/*Castanopsis*; MAP = 793.9–1389.4 mm, delimited by Arecaceae and *Ephedra*; MMaP = 141.5–245.2 mm, delimited by *Carya* and *Ephedra*; MMiP = 6.9–22.1 mm, delimited by *Carya* and *Pterocarya*.

The new parameters were compared with the current meteorological data in Wutu. The comparison revealed that the median values of MAT, MCMT, MAP, MMaP and MMiP were higher, and the median values of MWMT and DT were lower than today (Table D in S1 File). This indicates that the Early Eocene climate in Wutu was warmer and wetter, with weaker seasonality than today.

**Fig 8:**
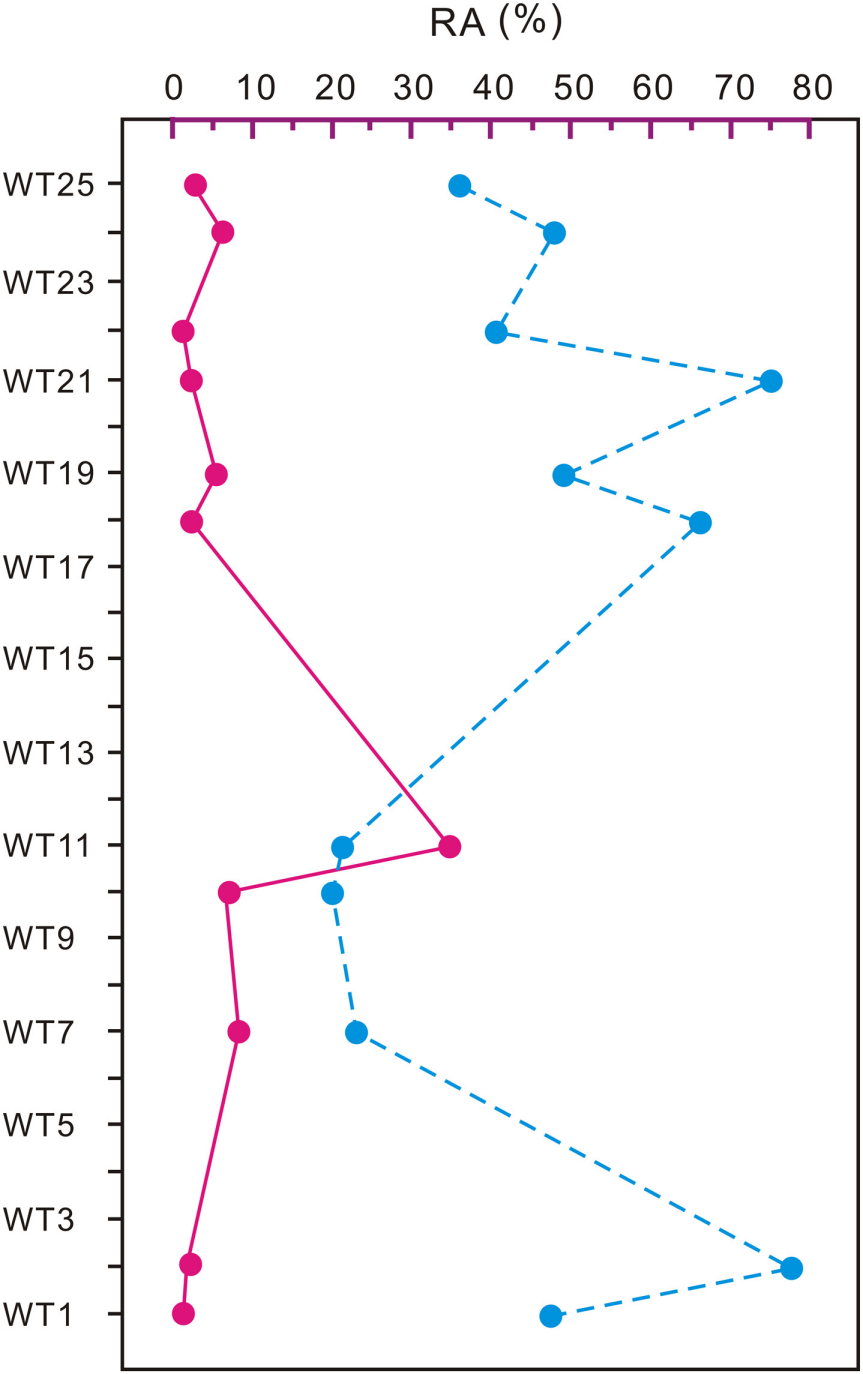
Changes in relative abundance (RA) of megathermic/mega-mesothermic (red line) and microthermic/meso-microthermic (blue line) elements.

The palaeoclimatic fluctuations were inferred from the changes of RA of megathermic/microthermic elements (Fig 8) and hygrophilous/xerophitous elements during the Periods 1–3. Period 1 was characterized by a warm temperate and humid climate. Period 2 was characterized by a relatively warmer and wetter climate, and Period 3 was cooler and drier again. Taken as a whole, the palynological data suggests that the Early Eocene climate in Wutu did not experience strong fluctuations during the three periods.

## Discussion

### The climate in East China during the last 65 million years

The palaeoclimate analysis developed from qualitative to quantitative in recent years. On the long-term scale, Mosbrugger et al. [41] studied the climatic changes of Central Europe during the last 45 million years, but more quantitative reconstructions of continental palaeoclimates need to be carried out. For now, the data on long-term climatic changes of East China are still missing. Unfortunately, no basin with the whole Cenozoic stratigraphic sequence like in Central Europe was found yet in East China, so 25 localities (including Wutu) from different latitudes were used here to reconstruct the Cenozoic palaeoclimate in East China (Fig 1). By comparing the palaeoclimatic parameters, this research represents an attempt in exploring the climatic changes in East China during the last 65 million years.

**Fig 9:**
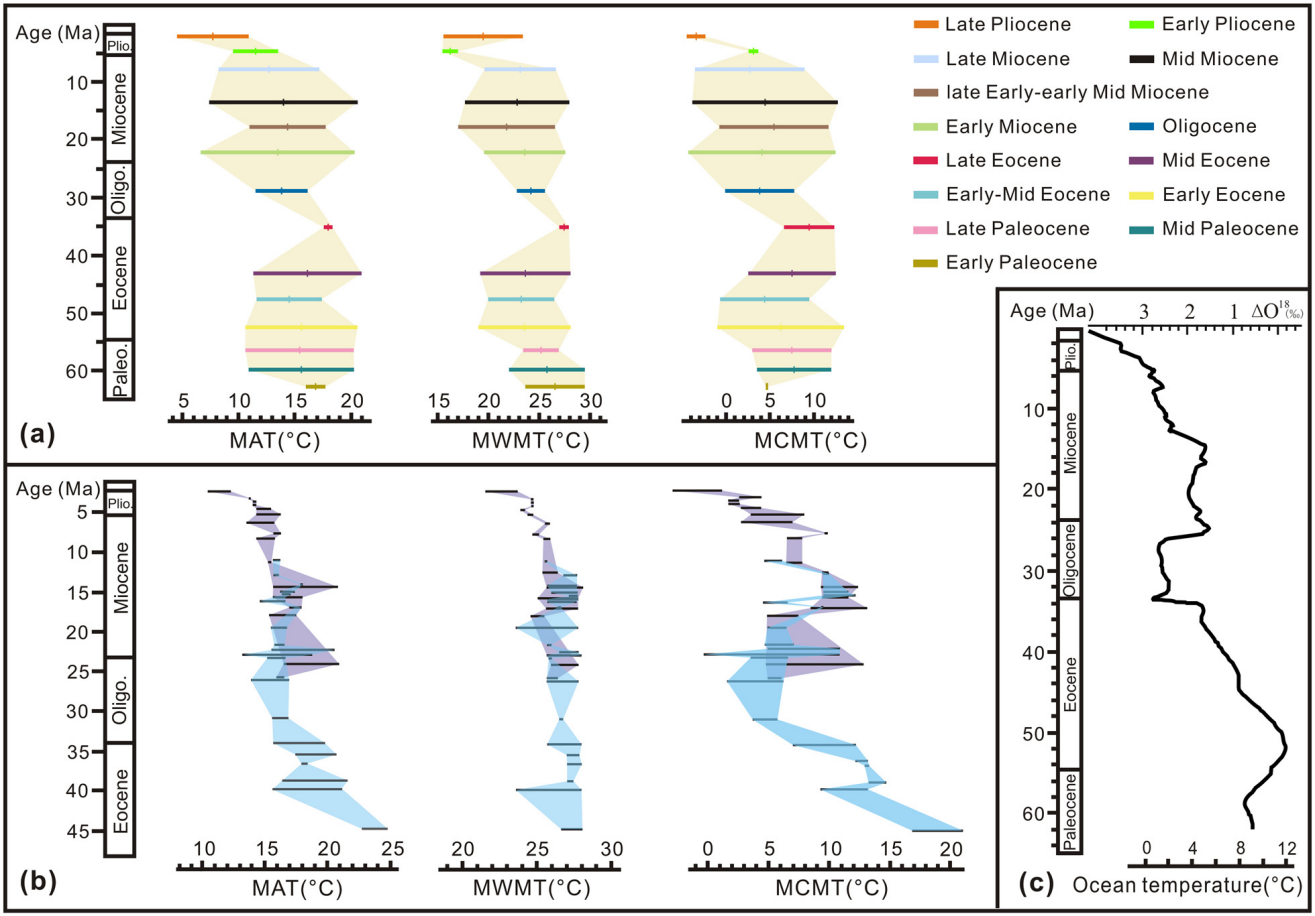
Evolution of the Cenozoic temperature curves. (a) Continental curves calculated by the coexistence approach (CA) for East China during the last 65 million years. (b) Continental curves calculated by the CA for Central Europe during the last 45 million years (from Mosbrugger et al. 2005). (c) Temperature change trends based on marine oxygene isotope record (from Zachos et al. 2001).

The climatic changes in East China during the Cenozoic may be illustrated by the comparison of climatic parameters obtained from 40 fossil floras [12, 13, 14, 16, 29, 32, 42, 43, 44] (including this study) (Table E in S1 File). Table F in S1 File shows the temperature parameters of Wutu and the published climatic data of the other 39 fossil floras. As obvious from Fig 1, these sites span approximately 30° latitudes. Hao et al. [13] obtained the latitudinal temperature gradients of 0.24 in the Paleocene, 0.1 in the Eocene, 0.45 in the Miocene and 0.55 in the Pliocene. Because the latitudinal temperature gradient in Oligocene is not available, the climatic parameters of the Paleocene, Eocene, Miocene and Pliocene were converted to 44.5° N (Shulan, Oligocene) through the palaeotemperature gradients. Table G in S1 File shows the comparison of climatic parameters after considering the latitudinal temperature gradients. The following three temperature curves of East China were obtained (Fig 9, Table H in S1 File):

1. MAT: Firstly, MAT curve showed a cooling period from the Early to Late Paleocene with median values of MAT decreasing by 1.4°C (from 16.9 to 15.5°C) (Table H in S1 File). Secondly, a warming trend from the Late Paleocene to Early Eocene is characterized by MAT increasing by 0.2°C (from 15.5 to 15.7°C). Thirdly, MAT curve showed a cooling period from the Early to Early-Middle Eocene with MAT decreasing by 1.2°C (from 15.7 to 14.5°C). Fourthly, MAT increased by 3.4°C from the Early-Middle to Late Eocene (from 14.5 to 17.9°C). Fifthly, MAT dropped by 4.4°C from the Late Eocene to Early Miocene (from 17.9 to 13.5°C). Sixthly, MAT increased by 1.3°C from the Early to late Early-early Middle Miocene (from 13.5 to 14.8°C). Finally, a cooling trend occurred from the late Early-early Middle Miocene to Late Pliocene, MAT dropped by 7.2°C (from 14.8 to 7.6°C). MAT curve showed a series of warming and cooling fluctuations. Taken as a whole, MAT curve indicated a general cooling trend in East China during the last 65 million years with MAT dropped by 9.3°C (from 16.9 to 7.6°C).
  This cooling trend coincided with the ocean temperature changes but with weaker amplitude (Fig 9) [1]. Based on the global marine oxygen isotope record, Zachos et al. [1] obtained the ocean water temperature curve. Although since the Early Oligocene the variability in oxygen isotope record mostly reflected the changes in Antarctica and Northern Hemisphere ice volume, it still reveals the global cooling trend [1].
2. MWMT: Firstly, MWMT dropped by 3.3°C from the Early Paleocene to Early-Middle Eocene (from 26.6 to 23.3°C). Secondly, it increased by 4.2°C from the Early-Middle to Late Eocene (from 23.3 to 27.5°C). Thirdly, it dropped by 5.6°C from the Late Eocene to late Early-early Middle Miocene (from 27.5 to 21.9°C). Fourthly, it increased by 1.3°C from the late Early-early Middle to Late Miocene (from 21.9 to 23.2°C). Fifthly, it dropped by 7.0°C from the Late Miocene to Early Pliocene (from 23.2 to 16.2°C). Finally, it increased by 3.3°C from the Early to Late Pliocene (from 16.2 to 19.5°C). Taken as a whole, MWMT curve suggested a general cooling trend during the whole Cenozoic with MWMT dropping by 7.1°C (from 26.6 to 19.5°C) (Fig 9).
3. MCMT: Firstly, MCMT increased by 2.9°C from the Early to Middle Paleocene (from 4.8 to 7.7°C). Secondly, MCMT decreased by 3.4°C from the Middle Paleocene to Early-Middle Eocene (from 7.7 to 4.3°C). Thirdly, it increased by 5.2°C from the Early-Middle to Late Eocene (from 4.3 to 9.5°C). Fourthly, it decreased by 5.6°C from the Late Eocene to Oligocene (from 9.5 to 3.9°C). Fifthly, it increased by 1.6°C from the Oligocene to late Early-early Middle Miocene (from 3.9 to 5.5°C). Sixthly, MCMT decreased by 2.8°C from the late Early-early Middle to Late Miocene (from 5.5 to 2.7°C). Then it increased by 0.3°C from the Late Miocene to Early Pliocene (from 2.7 to 3.0°C). Finally, it dropped by 6.4°C from the Early to Late Pliocene (from 3.0 to -3.4°C). MCMT curve suggested a general decreasing trend during the whole Cenozoic with MCMT dropping by 8.2°C (from 4.8 to -3.4°C) (Fig 9).

The comparison between the three temperature curves of East China suggests that during the last 65 million years, the changing trend of MCMT and MWMT coincided with MAT changes.

### Comparison with the Cenozoic climatic changes in Central Europe

The Cenozoic temperature curves of East China were compared with those from Central Europe [41]. It revealed that:

1. MAT: MAT curves exhibit the same trends but in different amplitudes. The decrease in MAT in East China was gentler than in Central Europe since the Early-Middle Eocene. MAT dropped by 6.9°C (from 14.5 to 7.6°C) in East China and by approximately 13°C (from 24 to 11°C) in Central Europe. The temperature was 3.4°C lower in East China than in Central Europe (7.6 vs. 11°C) during the Late Pliocene, while it was 9.5°C (14.5 vs. 24°C) lower in East China than in Central Europe during the Early-Middle Eocene (Fig 9).
2. MWMT: The decrease in MWMT in East China and that in Central Europe were more or less similar. MWMT dropped by 3.8°C (from 23.3 to 19.5°C) in East China and by 4.8°C (from 27.3 to 22.5°C) in Central Europe during the last 45 million years (Fig 9).
3. MCMT: The decrease in MCMT in East China was much gentler than in Central Europe. MCMT dropped by 7.7°C (from 4.3 to -3.4°C) in East China, but it dropped by approximately 20°C (from 19 to -1°C) in Central Europe during the last 45 million years. The difference of winter temperatures between East China and Central Europe was 2.4°C (-3.4 vs. -1°C) during the Late Pliocene, but it was 14.7°C (4.3 vs. 19°C) lower in East China than in Central Europe during the Early-Middle Eocene (Fig 9).

The Cenozoic cooling trend in East China was thus gentler than in Central Europe and mainly due to an obvious cooler climate in East China than the subtropical climate in Central Europe during the Early-Middle Eocene. This phenomenon may be related to the palaeogeography during the Eocene. Some authors have already pointed out the predominance of the large benthic foramifera *Nummulites* induced by the warm seawater all along the Tethys palaeomargins during the Eocene [45, 46]. Following global palaeogeographic reconstructions [47], East China was influenced by the northern Pacific oceanic circulation during the Early-Middle Eocene, while Europe was influenced by the warm Tethys Ocean causing the subtropical climate.

## Conclusions

The comprehensive palaeoecological and palaeoclimatic studies of the pollen samples from the Wutu Formation (Early Eocene) lead to the following conclusions:

1. The vegetation in Wutu in the Early Eocene time was composed of a mixed deciduous broad-leaved and coniferous forest growing under warm temperate condition.
2. Based on the palynological data, we reconstructed the climatic parameters of Wutu area in the Early Eocene by applying the CA method. They were MAT = 11.5–20.8°C, MWMT = 19.8–28.0°C, MCMT = -0.2–5.9°C, DT = 12.3–26.0°C, MAP = 793.9–1389.4 mm, MMaP = 141.5–245.2 mm and MMiP = 6.9–22.1 mm.
3. The climatic changes in East China during the Cenozoic were illustrated by comparing the climatic changes in Wutu and other 39 fossil floras in different ages. The temperature curves revealed that the climate in East China became gradually cooler during the last 65 million years, with MAT dropped by 9.3°C (from 16.9 to 7.6°C).
4. The cooling trend in East China (MAT dropped by 6.9°C) was gentler than in Central Europe (MAT dropped by approximately 13°C) during the last 45 million years. This phenomenon may be caused by the Early-Middle Eocene climate, which was much cooler in East China than in Central Europe. The MCMT was 14.7°C lower in East China than in Central Europe during the Early-Middle Eocene.

## Supporting Information

S1 File **Figure A, Coexistence intervals for all the parameters calculated by the CA.** The arrows show determination taxa for seven climatic parameters (1. *Pinus*; 2. *Picea*; 3. *Abies*; 4. Taxodiaceae; 5. *Ephedra*; 6. *Alnus*; 7. *Betula*; 8. *Castanopsis*; 9. *Tilia*; 10. *Quercus*; 11. *Corylus*; 12. *Juglans*; 13. *Ulmus*; 14. *Carya*; 15. *Pterocarya*; 16. *Ilex*; 17. *Artemisia*; 18. Arecaceae; 19. Aceraceae; 20. Poaceae; 21 .Juglandaceae; 22. Onagraceae; 23. Sapindaceae; 24. Euphorbiaceae; 25. Magnoliaceae; 26. Cyperaceae; 27. Umbelliferae; 28. Caprifolaceae; 29. Asteraceae; 30. Rosaceae; 31. Bignoniaceae; 32. Potamogetonaceae, 33. Chenopodiaceae). **Table A, Palynomorph percentages of Wutu and the comparison of palynological taxa between the whole palynoassemblage and palynological zones. Table B, List of the Early Eocene Wutu taxa grouped by ecological requirements and their relative abundance in palynological zones** (table style refers to Jiménez-Moreno, 2006 and Li et al., 2009). **Table C, The fossil palynomorph taxa used in coexistence approach (CA) along with their nearest living relatives (NLRs)** (Song 1999). **Table D, Comparison between the seven climatic parameters in the early Eocene of Wutu and the current meteorological data** (▲—median value of the climatic parameters; ■—mean value of the climatic parameters). **Table E, List of fossil localities in East China** (Site numbers as in Fig 1). **Table F, Comparison of climatic parameters of fossil localities in East China extended from the Early Paleocene to the Late Pliocene. Table G, After considering the latitudinal temperature gradient (correction to 44.5° N), comparison of climatic parameters of all the fossil localities. Table H, The Cenozoic temperature evolution of East China.**
(PDF)

## References

[1] Zachos J, Pagani M, Sloan L, Thomas E, Billups K. Trends, rhythms and aberrations in global climate 65 Ma to present. Science 2001; 292: 686–693. 11326091 10.1126/science.1059412

[2] Wing SL, Harrington GJ, Smith FA, Bloch JI, Boyer DM, Freeman KH. Transient floral change and rapid global warming at the Paleocene-Eocene boundary. Science. 2005; 310: 993–996. 16284173 10.1126/science.1116913

[3] Smith T, Rose KD, Gingerich PD. Rapid Asia—Europe—North America geographic dispersal of earliest Eocene primate Teilhardina during the Paleocene—Eocene Thermal Maximum. PNAS. 2006; 103: 11223–11227. 16847264 10.1073/pnas.0511296103 PMC1544069

[4] Wing SL, Alroy J, Hickey LJ. Plant and Mammal Diversity in the Paleocene to Early Eocene of the Bighorn Basin. Palaeogeography Palaeoclimatology Palaeoecology. 1995; 115: 117–155.

[5] Frederiksen NO. Upper Paleocene and lowermost Eocene angiosperm pollen biostratigraphy of the eastern Gulf Coast and Virginia. Micropaleontology. 1998; 44: 5–68.

[6] Wing SL, Harrington GJ. Floral response to rapid warming in the earliest Eocene and implications for concurrent faunal change. Paleobiology. 2001; 27: 539–563.

[7] Steurbaut E, Magioncalda R, Dupuis C, van Simaeys S, Roche E, Roche M. Palynology, paleoenvironments and organic carbon isotope evolution in lagoonal Paleocene-Eocene boundary settings in North Belgium. Geological Society of America Special Papers. 2003; 369: 291–317.

[8] Harrington GJ, Kemp SJ, Koch PL. Palaeocene-Eocene paratropical floral change in North America: responses to climate change and plant immigration. Journal of the Geological Society. 2004; 161: 173–184.

[9] Harrington GJ, Clechenko ER, Kelly DC. Palynology and organic-carbon isotope ratios across a terrestrial Palaeocene/Eocene boundary section in the Williston Basin, North Dakota, USA. Palaeogeography Palaeoclimatology Palaeoecology. 2005; 226: 214–232.

[10] Harrington GJ. Comparisons between Palaeocene-Eocene paratropical swamp and marginal marine pollen floras from Alabama and Mississippi, USA. Palaeontology. 2008; 51: 611–622.

[11] Collinson ME, Steart DC, Harrington GJ, Hooker JJ, Scott AC, Allen LO et al Palynological evidence of vegetation dynamics in response to palaeoenvironmental change across the onset of the Paleocene -Eocene Thermal Maximum at Cobham, Southern England. Grana. 2009; 48: 38–66.

[12] Yao YF, Bera S, Ferguson DK, Mosbrugger V, Paudayal KN, Jin JH, et al Reconstruction of paleovegetation and paleoclimate in the Early and Middle Eocene, Hainan Island, China. Climatic Change. 2009; 92: 169–189.

[13] Hao H, Ferguson DK, Feng GP, Ablaev A, Wang YF, Li CS. Early Paleocene vegetation and climate in Jiayin, NE China. Climatic Change. 2010; 99: 547–566.

[14] Wang Q, Ferguson DK, Feng GP, Ablaev AG, Wang YF, Yang J, et al Climatic change during the Palaeocene to Eocene based on fossil plants from Fushun, China. Palaeogeography Palaeoclimatology Palaeoecology. 2010; 295: 323–331.

[15] Meng QT, Liu ZJ, Bruch AA, Liu R, Hu F. Palaeoclimatic evolution during Eocene and its influence onoil shale mineralisation, Fushun basin, China. Journal of Asian Earth Science. 2012; 45: 95–105.

[16] Quan C, Liu YS, Utescher T. Paleogene temperature gradient, seasonal variation and climate evolution of northeast China. Palaeogeography Palaeoclimatology Palaeoecology. 2012; 313–314: 150–161.

[17] Yang ZJ. Cenozoic geology of Yidu, Changle and Linqu, Shandong Province. Bulletin of the Geological Society of China. 1936; 15: 171–187.

[18] Tong YS, Wang JW. Fossil Mammals from the Early Eocene Wutu Formation of Shandong Province (Palaeontologia Sinica, Whole Number 192, New Series C, Number 28). Science Press, Beijing; 2006.

[19] Chen L, Manchester SR, Chen ZD. Anatomically preserved seeds of Nuphar (Nymphaeaceae) from the early Eocene of Wutu, Shandong Province, China. American Journal of Botany. 2004; 91: 1265–1272. doi: 10.3732/ajb.91.8.1265 21653484 10.3732/ajb.91.8.1265

[20] Li Y, Smith T, Liu CJ, Awasthi N, Yang J, Wang YF et al Endocarps of Prunus (Rosaceae: Prunoideae) from the Early Eocene of Wutu, Shandong Province, China. Taxon 2011; 60: 555–564.

[21] Song ZC, Cao L, Li MY. Tertiary palynological assemblages from Shandong Province. Bulletin of the Nanjing Institute of Geology and Palaeontology, Chinese Academy of Sciences. 1964; 3: 179–290.

[22] Wang XM, Wang MZ, Zhang XQ. Eocene palynostratigraphy of Wutu, Shandong and its stratigraphical significance. Journal of Stratigraphy. 2005; 29: 22–27.

[23] Bloch JI, Fisher DC, Rose KD, Gingerich PD. Stratocladistic analysis of Paleocene Carpolestidae (Mammalia, Plesiadapiformes) with description of a new late Tiffanian genus. Journal of Vertebrate Paleontology. 2001; 21: 119–131.

[24] Zhao JS. Discussing Tertiary age problem based on microfossil and sporopollen of Wutu coal field. Shandong Geological Information. 1981; 4: 76–80.

[25] Moore PD, Webb JA, Collinson ME. Pollen analysis, 2nd ed Blackwell Scientific Publications 1991; 80: 127–131.

[26] Li XQ, Du NQ. The acid-alkali-free analysis of Quaternary pollen. Acta Botanica Sinica. 1999; 41: 782–784.

[27] Song ZC. Fossil spores and pollen of China, vol 1: the late Cretaceous and Tertiary spores and pollen. Science Press, Beijing; 1999.

[28] Ferguson DK, Zetter R, Paudayal KN. The need for the SEM in palaeopalynology. Comptes Rendus Palevol. 2007; 6: 423–430.

[29] Qin F, Ferguson DK, Zetter R, Wang YF, Syabryaj S, Li JF et al Late Pliocene vegetation and climate of Zhangcun region, Shanxi, North China. Global Change Biology. 2011; 17: 1850–1870.

[30] Zhang QQ, Ferguson DK, Mosbrugger V, Wang YF, Li CS. Vegetation and climatic changes of SW China in response to the uplift of Tibetan Plateau. Palaeogeography Palaeoclimatology Palaeoecology. 2012; 362–364: 23–36.

[31] Jimenez-Moreno G. Progressive substitution of a subtropical forest for a temperate one during the middle Miocene climate cooling in Central Europe according to palynological data from cores Tengelic-2 and Hidas-53 (Pannonian Basin, Hungary). Review of Palaeobotany and Palynology. 2006; 142: 1–14.

[32] Li JF, Ferguson DK, Yang J, Feng GP, Ablaev AG, Wang YF et al Early Miocene vegetation and climate in Weichang District, North China. Palaeogeography Palaeoclimatology Palaeoecology. 2009; 280: 47–63.

[33] Kershaw AP, Nix HA. Quantitative palaeoclimatic estimates from pollen data using bioclimatic profiles of extant data. Journal of Biogeography. 1988; 15: 589–602.

[34] Kershaw AP. A bioclimatic analysis of early to Middle Miocene brown coal floras, Latrobe Valley, South-Eastern Australia. Australian Journal of Botany. 1997; 45: 373–387.

[35] Mosbrugger V, Utescher T. The coexistence approach-a method for quantitative reconstruction of Tertiary terrestrial palaeoclimate data using plant fossils. Palaeogeography Palaeoclimatology Palaeoecology. 1997; 134: 61–86.

[36] Wu ZY, Ding TY. Seed Plants of Yunnan, China. Science and Technology Press, Kunming; 1999.

[37] IDBMC (Information Department of Beijing Meteorological Center). Land climate data of China (1951–1980) (part I–VI). China Meteorological Press, Beijing; 1984.

[38] Utescher T, Mosbrugger V. Palaeoflora Database. Available: http://www.palaeoflora.de. 2010.

[39] Grimm GW, Denk T. Reliability and resolution of the coexistence approach—a revalidation using modern-day data. Review of Palaeobotany and Palynology. 2012; 172: 33–47.

[40] Pross J, Klotz S, Mosbrugger V. Reconstructing palaeotemperatures for the Early and Middle Pleistocene using the mutual climatic range method based on plant fossils. Quaternary Science Review. 2000; 19: 1785–1799.

[41] Mosbrugger V, Utescher T, Dilcher DL. Cenozoic continental climate evolution of Central Europe. PNAS. 2005; 102: 14964–14969. 16217023 10.1073/pnas.0505267102 PMC1257711

[42] Yang J, Wang YF, Spicer RA, Mosbrugger V, Li CS, Sun QG. Climatic reconstruction at the Miocene Shanwang basin, China, using leaf margin analysis, CLAMP, coexistence approach, and overlapping distribution analysis. American Journal of Botany. 2007; 94: 599–608. doi: 10.3732/ajb.94.4.599 21636429 10.3732/ajb.94.4.599

[43] Li JF, Hu YQ, Ferguson DK, Wang YF, Li CS. An Early Pliocene lake and its surrounding vegetation in Zhejiang, East China. Journal of Paleolimnology. 2010; 43: 751–769.

[44] Liu YS, Utescher T, Zhou ZK, Sun BN. The evolution of Miocene climates in North China: preliminary results of quantitative reconstructions from plant fossil records. Palaeogeography Palaeoclimatology Palaeoecology. 2010; 304: 308–317.

[45] Racey A. A review of Eocene nummulite accumulations: Structure, Fomation and Reservior Potential. Journal of Petroleum Geology. 2001; 24: 79–100.

[46] Jorry SJ, Hasler CA, Davaud E. Hydrodynamic behaviour of Nummulites: implications for depositional models. Facies. 2006; 52: 221–235.

[47] Scotese CR. PALEOMAP project. Available: http://www.scotese.com/Default.htm. 2002.

[48] Shandong Provincial Institute of Land Surveying and Mapping. Atlas of Shandong Province. Sinomap Press, Beijing; 2014.

