# Correction: Evidence of a Cooler Continental Climate in East China during the Warm Early Cenozoic

**DOI:** 10.1371/journal.pone.0157802

**Published:** 2016-06-13

**Authors:** Qian-Qian Zhang, Thierry Smith, Jian Yang, Cheng-Sen Li

The following funding source is missing from the Funding section: the Belgian Federal Science Policy Office (BL/36/C54). The complete, correct funding statement is: This research was supported by the program of the Natural Science Foundation of China (NSFC) Nos. 41210001, 31370254 and 31300186, the National Basic Research Program of China No. 2014CB954201, the Belgian Federal Science Policy Office (BL/36/C54), the International S and T Cooperation Project of China No. 2009DFA32210, and the Basic Research Program of CAGS No. DZLXJK201405. The funders had no role in study design, data collection and analysis, decision to publish, or preparation of the manuscript.
