# Supplementary material for: Evidence of a Cooler Continental Climate in East China during the Warm Early Cenozoic
Source: PLoS One. 2016 May 19;11(5):e0155507. doi: 10.1371/journal.pone.0155507 (PMC4873231; doi:10.1371/journal.pone.0155507)
Supplement: S1 File — Figure A, Coexistence intervals for all the parameters calculated by the CA. The arrows show determination taxa for seven climatic parameters (1. Pinus; 2. Picea; 3. Abies; 4. Taxodiaceae; 5. Ephedra; 6. Alnus; 7. Betula; 8. Castanopsis; 9. Tilia; 10. Quercus; 11. Corylus; 12. Juglans; 13. Ulmus; 14. Carya; 15. Pterocarya; 16. Ilex; 17. Artemisia; 18. Arecaceae; 19. Aceraceae; 20. Poaceae; 21 .Juglandaceae; 22. Onagraceae; 23. Sapindaceae; 24. Euphorbiaceae; 25. Magnoliaceae; 26. Cyperaceae; 27. Umbelliferae; 28. Caprifolaceae; 29. Asteraceae; 30. Rosaceae; 31. Bignoniaceae; 32. Potamogetonaceae, 33. Chenopodiaceae). Table A, Palynomorph percentages of Wutu and the comparison of palynological taxa between the whole palynoassemblage and palynological zones. Table B, List of the Early Eocene Wutu taxa grouped by ecological requirements and their relative abundance in palynological zones (table style refers to Jiménez-Moreno, 2006 and Li et al., 2009). Table C, The fossil palynomorph taxa used in coexistence approach (CA) along with their nearest living relatives (NLRs) (Song 1999). Table D, Comparison between the seven climatic parameters in the early Eocene of Wutu and the current meteorological data (▲—median value of the climatic parameters; ■—mean value of the climatic parameters). Table E, List of fossil localities in East China (Site numbers as in Fig 1). Table F, Comparison of climatic parameters of fossil localities in East China extended from the Early Paleocene to the Late Pliocene. Table G, After considering the latitudinal temperature gradient (correction to 44.5° N), comparison of climatic parameters of all the fossil localities. Table H, The Cenozoic temperature evolution of East China. (PDF) [file pone.0155507.s001.pdf]

Fig. A

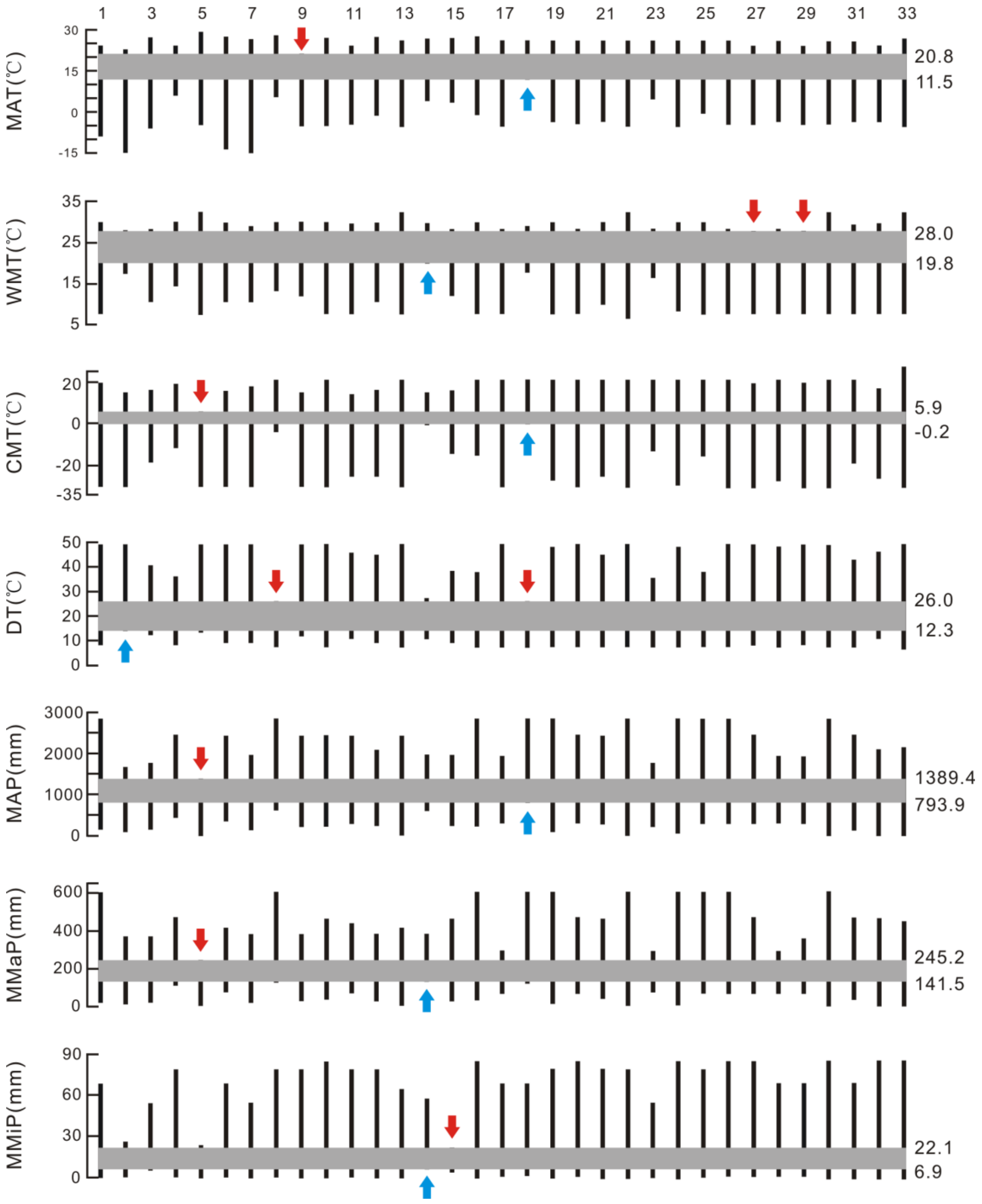

Fig. A Coexistence intervals for all the parameters calculated by the CA.

Table A Palynomorph relative abundance of Wutu whole section and palynological zones.

palynological taxa	Whole section	Zone No. 1	Zone No. 2	Zone No. 3	palynological taxa	Whole section	Zone No. 1	Zone No. 2	Zone No. 3
	(%)	(%)	(%)	(%)		(%)	(%)	(%)	(%)
**Gymnosperm**	**49.51**	**67.38**	**35.22**	**52.68**	*Ulmipollenites*	0.03	/	0.13	/
Pinuspollenites	41.54	55.91	21.50	47.68	*Tiliaepollenites*	0.03	/	0.13	/
Taxodiaceaepollenites	3.14	0.36	8.84	1.20	*Artemisiaepollenites*	0.03	/	0.13	/
Psophosphaera	2.76	7.89	1.58	2.46	Bignoniaceae	0.03	/	0.13	/
Ephedripites	1.71	2.87	3.17	0.93	*Corsinipollenites*	0.03	/	0.13	/
Abiespollenites	0.28	0.36	0.13	0.33	*Lonicerapollis*	0.03	/	0.13	/
Piceapollis	0.03	/	/	0.05	*Tricolporopollenites rosaeformis*	0.03	/	0.13	/
Ginkgoretectina	0.03	/	/	0.05	*Ilexpollenites*	0.03	/	/	0.05
**Angiosperm**	**48.36**	**30.82**	**60.95**	**45.85**	*Sapindaceidites*	0.03	/	/	0.05
Momipites coryloides	29.89	24.73	35.88	28.21	*Echitricoloorites*	0.03	/	/	0.05
Betulaceoipollenites	7.01	2.15	7.52	7.55	*Umbelliferaepites*	0.03	/	/	0.05
Castanopsis	3.17	1.43	7.65	1.59	*Chenopodipollis*	0.03	/	/	0.05
Tricolporopollenites	2.23	1.43	4.75	1.31	**Pteridopytes**	**1.99**	**1.79**	**3.69**	**1.31**
Alnipollenites	2.09	0.72	1.85	2.41	*Leiotriletes*	1.05	0.72	2.51	0.49
Juglanspollenites	1.08	/	0.13	1.64	*Pteris*	0.24	/	0.13	0.33
Pterocaryapollenites	1.05	/	0.40	1.48	Hemionitidaceae	0.21	0.36	0.40	0.11
Caryapollenites	0.31	0.36	0.13	0.38	Polypodiaceae	0.17	0.36	0.13	0.16
Momipites	0.28	/	0.26	0.33	Loxogrammaceae	0.10	/	/	0.16
Graminidites	0.21	/	0.40	0.16	*Subturma azonomonoletes*	0.07	0.36	0.13	/
Cyperaceaepollis	0.14	/	0.40	0.05	Dennstaedtiaceae	0.03	/	0.13	/
Euphorbiacites	0.10	/	0.26	0.05	Hymenophyllaceae	0.03	/	0.13	/
Palmaepollenites	0.10	/	0.13	0.11	*Selaginella*	0.03	/	0.13	/
Magnolipollis	0.07	/	0.26	/	*Osmunda*	0.03	/	/	0.05
Quercoidites	0.07	/	/	0.11	**Other elements**	**0.14**	**/**	**0.13**	**0.16**
Aceripollenites	0.07	/	/	0.11	Zygnemataceae	0.10	/	0.13	0.11
Potamogetonacidites	0.07	/	/	0.11	Pediastraceae	0.03	/	/	0.05

/, no pollen grain or spore of this taxon exists.

Table B List of the Early Eocene Wutu taxa grouped by ecological requirements and their relative abundance in palynological zones.

Zone No.	1(%)	2(%)	3(%)	Zone No.	1(%)	2(%)	3(%)
**Megathermic elements**				*Ulmipollenites*	/	0.13	/
Palmaepollenites	/	0.13	0.11	*Lonicerapollis*	/	0.13	/
Sapindaceidites	/	/	0.05	*Tiliaepollenites*	/	0.13	/
**Mega-mesothermic elements**				**Meso-microthermic elements**			
Taxodiaceaepollenites	0.36	8.84	1.20	*Pinuspollenites*	55.91	21.50	47.68
Castanopsis	1.43	7.65	1.59	**Microthermic elements**			
Magnolipollis	/	0.26	/	*Abiespollenites*	0.36	0.13	0.33
Euphorbiacites	/	0.26	0.05	*Piceapollis*	/	/	0.05
Bignoniaceae	/	0.13	/	**Non-significant elements**			
**Mesothermic elements**				*Tricolporopollenites rosaeformis*	/	0.13	/
Momipites coryloides	24.73	35.88	28.21	**Herbs and shrubs**			
Betulaceoipollenites	2.15	7.52	7.55	*Ephedripites*	2.87	3.17	0.93
Alnipollenites	0.72	1.85	2.41	*Graminidites*	/	0.40	0.16
Pterocaryapollenites	/	0.40	1.48	*Cyperaceaepollis*	/	0.40	0.05
Caryapollenites	0.36	0.13	0.38	*Potamogetonacidites*	/	/	0.11
Juglanspollenites	/	0.13	1.64	*Corsinipollenites*	/	0.13	/
Momipites	/	0.26	0.33	*Artemisiaepollenites*	/	0.13	/
Quercoidites	/	/	0.11	*Echitricoloorites*	/	/	0.05
Aceripollenites	/	/	0.11	*Umbelliferaepites*	/	/	0.05
Ginkgoretectina	/	/	0.05	*Chenopodipollis*	/	/	0.05
Ilexpollenites	/	/	0.05				

/, no pollen grain or spore of this taxon exists.

Table C The fossil palynomorph taxa used in CA along with their nearest living relatives (NLRs).

Fossil palynomorph taxa	NLRs
Pinuspollenites	*Pinus*
Abiespollenites	*Abies*
Piceapollis	*Picea*
Ephedripites	*Ephedra*
Taxodiaceaepollenites	Taxodiaceae
Ilexpollenites	*Ilex*
Palmaepollenites	Arecaceae
Sapindaceidites	Sapindaceae
Euphorbiacites	Euphorbiaceae
Bignoniaceae	Bignoniaceae
Castanopsis	*Castanopsis*
Magnolipollis	Magnoliaceae
Betulaceoipollenites	*Betula*
Momipites coryloides	*Corylus*
Ulmipollenites	*Ulmus*
Quercoidites	*Quercus*
Alnipollenites	*Alnus*
Caryapollenites	*Carya*
Tiliaepollenites	*Tilia*
Momipites	Juglandaceae
Juglanspollenites	*Juglans*
Pterocaryapollenites	*Pterocarya*
Aceripollenites	Aceraceae
Lonicerapollis	Caprifoliaceae
Tricolporopollenites rosaeformis	Rosaceae
Cyperaceaepollis	Cyperaceae
Echitricoloorites	Asteraceae
Artemisiaepollenites	*Artemisia*
Corsinipollenites	Onagraceae
Potamogetonacidites	Potamogetonaceae
Umbelliferaepites	Umbelliferae
Graminidites	Poaceae
Chenopodipollis	Chenopodiaceae

Table D Comparison between the seven climatic parameters in the Early Eocene of Wutu and the current meteorological data.

	Early Eocene ▲	Present ■
MAT(℃)	16.2	12.3
MWMT(℃)	23.9	25.9
MCMT(℃)	2.9	-3.2
DT(℃)	19.2	29.1
MAP(mm)	1091.7	671.5
MMaP(mm)	193.4	200.0
MMiP(mm)	14.5	7.8

Table E List of fossil localities in East China.

Period	Site	Location	Formation	Site coordinate	References
Late Pliocene	16	Zhangcun	Zhangcun	36.9 °N, 112.8 °E	Qin et al. 2011
Early Pliocene	24	Du’ao		29.3 °N, 121.5 °E	Li et al. 2010
Late Miocene	15	Huanghua		39.5 °N, 117.5 °E	Liu et al. 2010
	17	Jiyang	Minghuazhen (upper)	36.9 °N, 117.2 °E	Liu et al. 2010
	2	Huanan	Daotaiqiao	47 °N, 130 °E	Liu et al. 2010
Mid Miocene	20	Shanwang	Shanwang	36.5 °N, 118.7 °E	Yang et al. 2007
	9	Erlian	Tonggure	43.6 °N, 111.9 °E	Liu et al. 2010
	22	Tianchang	Yancheng	33 °N, 118 °E	Liu et al. 2010
	18	Bozhong	Minghuazhen (lower)	36.9 °N, 119 °E	Liu et al. 2010
	23	Zhoukou	Guantao	33.6 °N, 114.6 °E	Liu et al. 2010
	4	Jidong		45.2 °N, 131 °E	Liu et al. 2010
	2	Huanan	Daodaiqiao	47 °N, 130 °E	Liu et al. 2010
	11	Hunchun	Tumenzi	42.8 °N, 130 °E	Liu et al. 2010
late Early-early Mid Miocene	21	Lantian	Lengshuigou	34 °N, 108 °E	Liu et al. 2010
	21	Lantian	Gaoling	34 °N, 108 °E	Liu et al. 2010
	14	Shangdou		41.3 °N, 113.5 °E	Liu et al. 2010
Early Miocene	12	Weichang		42.1 °N, 117.8 °E	Li et al. 2009
	17	Jiyang	Guantao	36.9 °N, 117.2 °E	Liu et al. 2010
	14	Shangdou		41.3 °N, 113.5 °E	Liu et al. 2010
	8	Dunhua	Qiuligou	43.3 °N, 128.1 °E	Liu et al. 2010
Oligocene	7	Shulan	Shuiquliu	44.5 °N, 126.9 °E	Quan et al. 2012
Late Eocene	13	Fushun	Gengjiajie	41.8 °N, 123.9 °E	Quan et al. 2012
	11	Hunchun	Hunchun (upper)	42.8 °N, 130.3 °E	Quan et al. 2012
Mid Eocene	13	Fushun	Jijuntun	41.8 °N, 123.9 °E	Wang et al. 2010
	13	Fushun	Xilutian	41.8 °N, 123.9 °E	Wang et al. 2010
	10	Huadian	Huadian	42.9 °N, 126.7 °E	Quan et al. 2012
	6	Mudanjiang	Huanghua	44.6 °N, 129.4 °E	Quan et al. 2012
	7	Shulan	Jishu	44.5 °N, 126.9 °E	Quan et al. 2012
	3	Yilan	Dalianhe	46.1 °N, 129.3 °E	Quan et al. 2012
	11	Hunchun	Hunchun (lower)	42.8 °N, 130.3 °E	Quan et al. 2012
Early-Mid Eocene	25	Changchang	Changchang	19.6 °N, 110.4 °E	Yao et al. 2009
Early Eocene	19	Wutu	Wutu	36.6 °N, 118.9 °E	This study
	13	Fushun	Guchengzi	41.8 °N, 123.9 °E	Wang et al. 2010
	7	Shulan	Bangchuigou	44.5 °N, 126.9 °E	Quan et al. 2012
	5	Hualin	Bahuli	44.8 °N, 129.8 °E	Quan et al. 2012
	3	Yilan	Xin'ancun	46.1 °N, 129.3 °E	Quan et al. 2012
Late Paleocene	13	Fushun	Lizigou	41.8 °N, 123.9 °E	Wang et al. 2010
Mid Paleocene	13	Fushun	Laohutai	41.8 °N, 123.9 °E	Wang et al. 2010
	1	Wuyun	Wuyun (upper)	49.3 °N, 129.5 °E	Hao et al. 2010
Early Paleocene	1	Wuyun	Wuyun (lower)	49.3 °N, 129.5 °E	Hao et al. 2010

Table F Comparison of climatic parameters of fossil localities in East China extended from the Early Paleocene to the Late Pliocene.

Period	Location	Formation	MAT	MWMT	MCMT
Late Pliocene	Zhangcun	Zhangcun	8.5-15.1	19.8-27.5	-0.3-2.0
Early Pliocene	Du’ao		18.1-22.0	23.8-25.4	10.7-12.1
Late Miocene	Huanghua		15.7-16.1	24.7-24.9	5-7.1
	Bozhong	Minghuazhen (upper)	11.6-18.4	23-28.2	-0.3-12.5
	Huanan	Daotaiqiao	14-16.1	24.7-25.6	-0.5-7.1
Mid Miocene	Shanwang	Shanwang	10.9-14.5	21.9-25.0	-0.5-3.3
	Erlian	Tonggure	13.3-21.1	24-28.3	-0.1-13.3
	Tianchang	Yancheng	15.7-16.1	23-25.6	3.8-7.8
	Bozhong	Minghuazhen (lower)	13.6-18.4	23.6-28.2	1.8-12.5
	Zhoukou	Guantao	12.5-16.1	24.9-25.6	1.7-7.1
	Jidong		11.5-15.8	23.0-25.6	1.7-5.6
	Huanan	Daodaiqiao	14-16.1	24.7-25.6	-0.5-6.2
	Hunchun	Tumenzi	16.5	27.3-27.4	5.5-7.1
late Early-early Mid Miocene	Lantian	Lengshuigou	15.7-20.8	21.7-28.1	3.8-13.3
	Lantian	Gaoling	16.8-18.4	23-28.1	10.6-12.5
	Shangdou		13.9-19.2	25.7-28.1	2.2-13.3
Early Miocene	Weichang	Hannuoba	7.8-14.9	23.5-25.4	-3.0-5.9
	Jiyang	Guantao	11.5-20.8	23.0-28.1	-1.0-13.3
	Shangdou		11.5-20.8	23.0-28.1	1.7-13.3
	Dunhua	Qiuligou	15.7-20.8	28.0-28.1	5.5-13.3
Oligocene	Shulan	Shuiqulju	11.6-16.1	22.8-25.6	-0.1-7.8
Late Eocene	Fushun	Gengjiajie	17.9-18.4	27.3-28.1	7.0-12.5
	Hunchun	Hunchun (upper)	17.9-18.4	27.3-28.1	7.0-12.5
Mid Eocene	Fushun	Jijuntun	14.8-20.9	19.4-24.7	9.1-12.6
	Fushun	Xilutian	11.5-20.9	22.5-26.6	3.6-12.6
	Huadian	Huadian	15.6-18.4	24.7-27.9	3.8-12.5
	Mudanjiang	Huanghua	17.9-18.3	24.7-27.7	7.0-10.2
	Shulan	Jishu	15.6-18.4	24.7-25.0	7.0-7.8
	Yilan	Dalianhe	13.3-20.8	24.7-27.9	2.2-12.5
	Hunchun	Hunchun (lower)	16.5-18.4	27.3-27.9	6.6-7.8
Early-Mid Eocene	Changchang	Changchang	14.2-19.8	22.5-29.1	1.7-11.9
Early Eocene	Wutu	Wutu	11.5-20.8	19.8-28.0	-0.2-5.9
	Fushun	Guchengzi	14.8-21.0	19.4-24.7	9.1-13.9
	Shulan	Bangchuigou	15.2-18.4	24.0-27.9	6.6-12.5
	Hualin	Bahuli	13.6-18.4	23.6-28.1	3.7-12.5
	Yilan	Xin'ancun	17.9-18.4	27.3-27.9	7.0-12.5
Late Paleocene	Fushun	Lizigou	11.3-20.9	23.9-27.5	3.6-12.6
Mid Paleocene	Fushun	Laohutai	11.5-20.9	22.5-28.2	3.6-12.6
	Wuyun	Wuyun (upper)	14.8-16.6	23.9-28.3	3.6-3.6
Early Paleocene	Wuyun	Wuyun (lower)	14.8-16.6	22.5-28.3	3.6-3.6

Table G After considering the latitudinal temperature gradient (correction to 44.5 °N), comparison of climatic parameters of all the fossil localities.

Period	Location	Formation	MAT	MWMT	MCMT
Late Pliocene	Zhangcun	Zhangcun	4.3-10.9	15.6-23.3	-4.5--2.2
Early Pliocene	Du'ao		9.7-13.6	15.4-17.0	2.3-3.7
Late Miocene	Huanghua		13.4-13.8	22.4-22.6	2.7-4.8
	Bozhong	Minghuazhen (upper)	8.2-15.0	19.6-24.8	-3.7-9.1
	Huanan	Daotaiqiao	15.1-17.2	25.8-26.7	0.6-8.2
Mid Miocene	Shanwang	Shanwang	7.3-10.9	18.3-21.4	-4.1--0.3
	Erlian	Tonggure	12.9-20.7	23.6-27.9	-0.5-12.9
	Tianchang	Yancheng	10.5-10.9	17.8-20.4	-1.4-2.6
	Bozhong	Minghuazhen (lower)	10.2-15.0	20.2-24.8	-1.6-9.1
	Zhoukou	Guantao	7.6-11.2	20.0-20.7	-3.2-2.2
	Jidong		11.8-16.1	23.3-25.9	2.0-5.9
	Huanan	Daodaiqiao	15.1-17.2	25.8-26.7	0.6-7.3
	Hunchun	Tumenzi	15.7	26.5-26.6	4.7-6.3
late Early-early Mid Miocene	Lantian	Lengshuigou	11.0-16.1	17.0-23.4	-0.9-8.6
	Lantian	Gaoling	12.1-13.7	18.3-23.4	5.9-7.8
	Shangdou		12.5-17.8	24.3-26.7	0.8-11.9
Early Miocene	Weichang	Hannuoba	6.7-13.8	22.4-24.3	-4.1-4.8
	Jiyang	Guantao	8.1-17.4	19.6-24.7	-4.4-9.9
	Shangdou		10.1-19.4	21.6-26.7	0.3-11.9
	Dunhua	Qiuligou	15.2-20.3	27.5-27.6	5.0-12.8
Oligocene	Shulan	Shuiquliu	11.6-16.1	22.8-25.6	-0.1-7.8
Late Eocene	Fushun	Gengjiajie	17.6-18.1	27.0-27.8	6.7-12.2
	Hunchun	Hunchun (upper)	17.7-18.2	27.1-27.9	6.8-12.3
Mid Eocene	Fushun	Jijuntun	14.5-20.6	19.1-24.4	8.8-12.3
	Fushun	Xilutian	11.2-20.6	22.2-26.3	3.3-12.3
	Huadian	Huadian	15.4-18.2	24.5-27.7	3.6-12.3
	Mudanjiang	Huanghua	17.9-18.3	24.7-27.7	7.0-10.2
	Shulan	Jishu	15.6-18.4	24.7-25.0	7.0-7.8
	Yilan	Dalianhe	13.5-21.0	24.9-28.1	2.4-12.7
	Hunchun	Hunchun (lower)	16.3-18.2	27.1-27.7	6.4-7.6
Early-Mid Eocene	Changchang	Changchang	11.7-17.3	20.0-26.6	-0.8-9.4
Early Eocene	Wutu	Wutu	10.7-20.0	19.0-27.2	-1.0-5.1
	Fushun	Guchengzi	14.5-20.7	19.1-24.4	8.8-13.6
	Shulan	Bangchuigou	15.2-18.4	24.0-27.9	6.6-12.5
	Hualin	Bahuli	13.6-18.4	23.6-28.1	3.7-12.5
	Yilan	Xin'ancun	18.1-18.6	27.5-28.1	7.2-12.7
Late Paleocene	Fushun	Lizigou	10.7-20.3	23.3-26.9	3.0-12.0
Mid Paleocene	Fushun	Laohutai	10.9-20.3	21.9-27.6	3.0-12.0
	Wuyun	Wuyun (upper)	16.0-17.8	25.1-29.5	4.8-4.8
Early Paleocene	Wuyun	Wuyun (lower)	16.0-17.8	23.7-29.5	4.8-4.8

Table H The Cenozoic temperature evolution of East China.

Period	MAT<br>min-max (mid)	MWMT<br>min-max (mid)	MCMT<br>min-max (mid)
Late Pliocene	4.3-10.9 (7.6)	15.6-23.3 (19.5)	-4.5--2.2 (-3.4)
Early Pliocene	9.7-13.6 (11.7)	15.4-17.0 (16.2)	2.3-3.7 (3.0)
Late Miocene	8.2-17.2 (12.7)	19.6-26.7 (23.2)	-3.7-9.1 (2.7)
Mid Miocene	7.3-20.7 (14.0)	17.8-27.9 (22.9)	-4.1-12.9 (4.4)
late Early-early Mid Miocene	11.0-17.8 (14.8)	17.0-26.7 (21.9)	-0.9-11.9 (5.5)
Early Miocene	6.7-20.3 (13.5)	19.6-27.6 (23.6)	-4.4-12.8 (4.2)
Oligocene	11.6-16.1 (13.9)	22.8-25.6 (24.2)	-0.1-7.8 (3.9)
Late Eocene	17.6-18.2 (17.9)	27.0-27.9 (27.5)	6.7-12.3 (9.5)
Mid Eocene	11.2-21.0 (16.1)	19.1-28.1 (23.6)	2.4-12.7 (7.6)
Early-Mid Eocene	11.7-17.3 (14.5)	20.0-26.6 (23.3)	-0.8-9.4 (4.3)
Early Eocene	10.7-20.7 (15.7)	19.0-28.1 (23.6)	-1.0-13.6 (6.3)
Late Paleocene	10.7-20.3 (15.5)	23.3-26.9 (25.1)	3.0-12.0 (7.5)
Mid Paleocene	10.9-20.3 (15.6)	21.9-29.5 (25.7)	3.4-12.0 (7.7)
Early Paleocene	16.0-17.8 (16.9)	23.7-29.5 (26.6)	4.8-4.8 (4.8)
